# Exploring the most demanding scenarios in elite youth basketball: a comprehensive analysis across playing positions and time windows

**DOI:** 10.5114/biolsport.2025.148537

**Published:** 2025-04-14

**Authors:** Yannis Irid, Julian Hutin, Jean-François Toussaint, Adrien Sedeaud

**Affiliations:** 1 Université Paris Cité, IRMES – UPR 7329, Institut de Recherche Médicale et d’Épidémiologie du Sport, France; 2 Institut National du Sport, de l’Expertise et de la Performance (INSEP), Paris, France; 3 Fédération Française de Basketball, Paris, France; 4 Centre d’Investigations en Médecine du Sport, CIMS Hôtel-Dieu, Assistance Publique – Hôpitaux de Paris, Paris, France

**Keywords:** Basketball, Youth athletes, Player monitoring, Game analysis, Peak demands, Local positioning system

## Abstract

This study aimed to examine the Most Demanding Scenarios (MDS) in elite youth basketball players, focusing on position-specific differences across various time windows. Data were collected from 31 players (20 males, 11 females) across two seasons during 40 official games using a 20-Hz Local Positioning System and 100-Hz accelerometer. Metrics included total distance, high-speed running distance, accelerations, decelerations, jumps, and changes of direction. Peak Demands (PD), High-Intensity Periods (HIP), and Very High-Intensity Periods (VHIP) were calculated using rolling averages over 10, 30, 60, and 120-second windows. Mixed linear models compared PD across time intervals and between playing positions (frontcourt vs. backcourt). Shorter time windows showed higher relative peak values for both males and females. Relative distances declined with longer intervals, from 251.34 ± 23.46 m · min^−1^ (10 s) to 113.61 ± 13.52 m · min^−1^ (120 s) for males (p < 0.001; d = 7.20, nearly perfect effect), and from 237.37 ± 24.16 m · min^−1^ to 114.52 ± 11.7 m · min^−1^ for females (p < 0.001; d = 6.47, nearly perfect effect). Male backcourt players (BC) had higher PD than frontcourt players (FC) across most variables and windows, except for changes of direction. Female BC showed significantly higher PD in acceleration (10.26 ± 2.28 m vs. 9.07 ± 2.83 m; p = 0.04; d = -0.45, small effect) and deceleration distance (11.9 ± 2.39 m vs. 10.12 ± 3.9 m; p = 0.02; d = -0.53, small effect) over 120 s. Additionally, male FC were more frequently exposed to HIP over 10 s (p = 0.011; d = 0.20, trivial effect) and VHIP over 30 s (p = 0.001; d = 0.26, small effect) for distance covered, whereas female BC consistently demonstrated more frequent passages in VHIP for sprint durations across all time windows. These findings highlight significant position-specific differences in the MDS of elite youth basketball players. Understanding these demands emphasizes the need for tailored, position-specific training and conditioning programs to optimize performance.

## INTRODUCTION

Basketball is known for its intermittent and high-intensity nature, involving a complex blend of aerobic and anaerobic activities [1]. Players must perform various movements, such as accelerations, decelerations, changes of direction, jumps, and running, all within a single game [2]. The fluctuating intensity and diverse movement patterns inherent to this sport necessitate a comprehensive analysis of the performance demands placed upon players to facilitate their development and wellness [3].

Technological advancements have transformed the landscape of sports science, especially in monitoring the physical demands of basketball players [4]. The integration of electronic performance tracking systems (EPTS) such as Inertial Measurement Units (IMU) and Local Positioning Systems (LPS) has become commonplace in professional team sports seeking to obtain a competitive advantage [5]. This data-driven approach supports the decision-making process for prescribing and manipulating training loads [6]. Although technological advancements have greatly improved our ability to monitor and analyze the physical demands of basketball, there is currently a lack of alignment in practices and methodological frameworks in specific research [4]. This is particularly evident in the reliance on average values to represent an athlete’s performance and workload.

Traditional approaches, which focus on average values, drastically underestimate and may fail to capture the peak intensities and nuanced demands experienced during critical moments of play [7–9]. Consequently, quantifying the Most Demanding Scenarios (MDS) became of a paramount importance in basketball [10]. The MDS in basketball refers to periods characterized by maximal or near-maximal physical demands exceeding 80% of the player’s maximum intensity occurring within a specific timeframe [11]. These scenarios are subdivided into peak demand (PD), which is the highest intensity activity for a specific variable across a specified timeframe of interest [11], while high intensity (HIP) and very high intensity periods (VHIP) are defined as those covering intensity levels of 80–90% and > 90% of PD, respectively [11]. These scenarios reflect the actual stresses that players face and are critical for preparing athletes to withstand and excel during the most challenging phases of competition [7, 12]. A growing body of literature supports the idea that understanding MDS is crucial for optimizing training and performance strategies [7, 13, 14]. Furthermore, several studies have analyzed the impact of contextual variables on PD, including match timing, age, type of activity (e.g., training or match), final score, playing positions, playing time (top player, core player, and occasional player), team venue (home, away, or neutral), score outcome (win vs. loss), match nature (official or non-official), and their evolution across game quarters [10, 15]. Moreover, a variety of time windows (15, 30, 45 seconds, or 1, 2, 3, 4, 5, and 10 minutes) and variables are currently utilized to identify the MDS, with moving averages appearing to be the most suitable method for identifying PD [16]. Despite extensive research on the analysis of MDS in basketball competition [7, 9, 13, 17–22], their characterization remains incomplete. For instance, a recent review noted that while basketball studies often focus on PD, additional components of MDS, such as HIP, VHIP, and Worst-Case Scenarios (WCS), remain underexplored [11]. This highlights the necessity for a more detailed examination of MDS to better comprehend the precise physical constraints imposed on players. Finally, while the available research on MDS in male basketball is extensive, there is a significant gap in the literature regarding comprehensive female physical demands. Previous research has exclusively focused on male athletes, with less emphasis on understanding the physical demands placed on female players [23–25]. This is a significant oversight, given the differences in physiological and metabolic demands between male and female athletes, which may impact our understanding of the physical constraints experienced by female basketball players [24].

Therefore, the aim of this study is to provide a comprehensive characterization of MDS in elite youth basketball players. The objectives are to: (1) investigate MDS encountered by both male and female players, (2) classify MDS into subcategories (PD, HIP, and VHIP), and (3) determine the frequency of these periods across playing positions. By achieving these objectives, our findings aim to support the development of tailored, position-specific training programs that optimize performance for both male and female athletes.

## MATERIALS AND METHODS

### Subjects

Data were collected from 31 elite youth basketball players (Male: n = 20; height: 199.83 ± 5.16 cm; mass: 86.13 ± 10.13 kg; age: 17.25 ± 0.62 years; Female: n = 11; height: 182 ± 8 cm; mass: 72.25 ± 11.08 kg; age: 17.42 ± 0.67 years) from the French Institute of Sport (INSEP) teams over two consecutive seasons (2022–2023 and 2023–2024) during 40 official matches (males: n = 27; females: n = 13), resulting in 683 player-match samples (males: n = 540; females: n = 143). INSEP annually selects the top 50 French prospects (25 males, 25 females) aged 15 to 18, providing a high-performance environment aligned with recognized criteria for defining elite athletes [26, 27]. These players represent France in U16, U17 and U18 international competitions and compete in national professional leagues (males in NM1 [3^rd^ Division], females in LF2 [2^nd^ Division]) to accelerate their development at the senior competitive levels. Throughout the data collection period, players engaged in an average of 15.5 h of training per week, comprising 7 basketball sessions and 5 strength and conditioning sessions. Participants were categorized into two positional groups – Backcourt (Point Guards, Shooting Guards; males: n = 11, females: n = 4) and Frontcourt (Small Forwards, Power Forwards and Centers; males: n = 9, females: n = 7) – to reflect the evolving, position-specific demands of modern basketball [4].

### Ethics Statement

The collection of this data complies with the General Data Protection Regulations (GDPR) established by the European Union. The study was supervised and developed by the IRMES scientific committee. Ethical approval for the study protocol was obtained from the ethics panel of the Scientific, Medical, and Training Council (CSMF) at INSEP.

### Data Collection

Players’ activities during every home game were recorded using a 20-Hz Local Positioning System (LPS) and 100-Hz embedded accelerometer devices (Kinexon, Kinexon GMBH, Munich, Germany) [28]. The Kinexon Ultrawide Band (UWB) system consisted of 14 antennas positioned around the basketball court at the same heights, with players wearing tags on the upper back using manufacturer-provided harnesses. Signals were transmitted to the antennas using UWB technology in the 4.25–7.25 GHz frequency range. Player positions were calculated using proprietary algorithms based on Time Difference of Arrival, Two-Way Ranging, and Angle of Arrival methods [28]. The validity of the Kinexon LPS device has been established, showing a small standardized typical error of estimate (from 0.06 to 0.48) compared with 3-dimensional motion capture (Vicon) for sprint, lateral, and specific handball movements [28, 29]. Additionally, the interunit coefficient of variation ranged from 2.1 to 9.2% [29]. All official matches were played on the same court under similar environmental conditions. Players were continuously monitored during each match, with data analysis limited to active play periods, excluding rest intervals and substitutions. Due to the permanent installation of the monitoring system at the INSEP facility, data from away official matches were not included. Only data from players who participated for at least three minutes of live play within the same quarter were included to mitigate performance variations associated with limited playing time [30, 31]. The eight physical variables selected to describe match activities were: total distance covered (m), time spent running at high intensity (> 18 km · h^−1^) [32], the number of accelerations and decelerations (> 2 m · s^−2^), distance covered at high-intensity accelerations (> 2 m · s^−2^) and decelerations (< -2 m · s^−2^), the number of high-intensity jumps (> 0.40 m), and the number of high-intensity changes of direction (> 90° and < -2 m · s^−2^).

### Data Processing

**FIG. 1:**
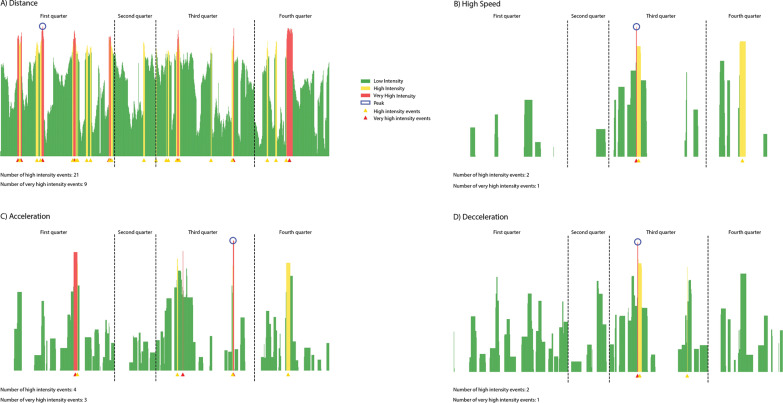
Illustration of the peak demands (PD), high intensity (HIP), and very high intensity periods (VHIP), for distance, accelerations, decelerations, and high-speed efforts, for one player over one game.

A rolling average was applied to each player’s data, obtained directly from the Kinexon API, over 10-, 30-, 60-, and 120-second windows (in 1-second intervals). The data set included position, speed, acceleration, and jump metrics. From these inputs, additional variables such as decelerations, changes of direction, and distance covered were independently calculated before applying the rolling averages. Rolling averages were reset at the end of each quarter and resumed at the beginning of the next. The maximum value of each variable within each time window was recorded as the PD. Thresholds were established to identify HIP and VHIP: 80–90% and > 90% of the PD, respectively [11]. The final step was to re-conduct a rolling average and record all sequences within the defined thresholds for each variable. Using moving averages with a 1-second step, the number of non-consecutive sequences per quarter within these thresholds was counted to describe the frequency of HIP and VHIP (Figure 1). Rolling averages are commonly used in team sports to calculate PD during predefined time epochs [13, 16, 22, 33]. In each time epoch, the peak values of the selected physical demand measures were recorded independently. The selected time epochs are based on studies indicating that approximately 90% of live-time actions in basketball last less than 80 seconds, with about 17% and 26% near 30 and 60 seconds, respectively, and actions exceeding 180 seconds being rare [34].

### Statistical Analysis

Data are presented as mean ± standard deviation (SD). Prior to conducting the analyses, the normality of residuals was assessed using the Shapiro-Wilk test to ensure that assumptions for linear mixed modeling were met. To compare PD across different time windows, variables were expressed relative to the window duration (in minutes). Linear mixed models were then employed to compare PD between time epochs (10 s, 30 s, 60 s, and 120 s), with time epoch as the fixed effect (4 levels) and matches as a random effect to account for repeated measures across different games. Linear mixed models were also used to compare PD and the frequency of HIP and VHIP between playing positions in both male and female players, with position as the fixed effect (2 levels) and matches as a random effect.

All statistical analyses were performed using Python (Version 3.12.5), leveraging the ‘statsmodels’ package for linear mixed models and ‘scipy’ for normality testing. Effect sizes (ES) (Cohen’s d) were calculated to provide a clearer interpretation of the magnitude of observed differences. The ES were interpreted as follows: ≤ 0.2, trivial; > 0.2, small; > 0.6, moderate; > 1.2, large; > 2.0, very large; and > 4.0, nearly perfect [35]. Statistical significance was set at *p* < 0.05.

## RESULTS

### Time Epoch Impact

Descriptive values for the relative PD of male and female teams over different time epochs (10 s, 30 s, 60 s, 120 s) are presented in Table 1. The results revealed that shorter epochs indicated significantly higher values for both males and females compared to longer intervals. For males, the relative distances covered (m · min^−1^) declined from 251.34 ± 23.46 m · min^−1^ over 10 s to 113.61 ± 13.52 m · min^−1^ over 120 s, reflecting a nearly perfect difference (p < 0.001; d = 7.20). Similarly, the relative number of accelerations and decelerations (n · min^−1^) followed the same trend, decreasing from 14.58 ± 3.12 n · min^−1^ to 3.1 ± 0.74 n · min^−1^ for accelerations (p < 0.001, d = 5.08), and from 14.1 ± 2.94 n · min^−1^ to 2.86 ± 0.77 n · min^−1^ for decelerations (p < 0.001, d = 5.11).

**Table 1:** Relative peak demands of male and female players over different time epochs (mean ± SD)

Team	Variable	Time windows			

		10 s	30 s	60 s	120 s
Male	Distance (m · min^−1^)	251.34 ± 23.46^b,c,d^	175.74 ± 15.96^a,c,d^	142.51 ± 11.69^a,b,d^	113.61 ± 13.52^a,b,c^
	Accelerations count (n · min^−1^)	14.58 ± 3.12^b,c,d^	7.18 ± 1.60^a,c,d^	4.58 ± 1.08^a,b,d^	3.1 ± 0.74^a,b,c^
	Accelerations distance (m · min^−1^)	38.34 ± 8.16^b,c,d^	16.68 ± 3.98^a,c,d^	10.34 ± 2.69^a,b,d^	6.69 ± 1.74^a,b,c^
	Decelerations count (n · min^−1^)	14.1 ± 2.94^b,c,d^	6.84 ± 1.54^a,c,d^	4.34 ± 1.13^a,b,d^	2.86 ± 0.77^a,b,c^
	Decelerations distance (m · min^−1^)	39.01 ± 9.00^b,c,d^	16.74 ± 4.16^a,c,d^	10.28 ± 2.85^a,b,d^	6.52 ± 1.86^a,b,c^
	Sprint duration (s · min^−1^)	21.48 ± 5.64^b,c,d^	8.34 ± 2.78^a,c,d^	4.77 ± 1.62^a,b,d^	2.74 ± 0.91^a,b,c^
	Jumps (n · min^−1^)	7.56 ± 3.66^b,c,d^	2.96 ± 1.58^a,c,d^	1.64 ± 0.88^a,b,d^	1.0 ± 0.56^a,b,c^
	Change of directions (n · min^−1^)	10.2 ± 3.84^b,c,d^	3.76 ± 1.42^a,c,d^	2.06 ± 0.75^a,b,d^	1.19 ± 0.43^a,b,c^

Female	Distance (m · min^−1^)	237.37 ± 24.16^b,c,d^	168.84 ± 13.88^a,c,d^	139.78 ± 12.42^a,b,d^	114.52 ± 11.7^a,b,c^
	Accelerations count (n · min^−1^)	12.99 ± 2.24^b,c,d^	6.19 ± 1.26^a,c,d^	3.73 ± 0.94^a,b,d^	2.44 ± 0.65^a,b,c^
	Accelerations distance (m · min^−1^)	30.65 ± 7.35^b,c,d^	12.85 ± 3.52^a,c,d^	7.4 ± 2.07^a,b,d^	4.78 ± 1.33^a,b,c^
	Decelerations count (n · min^−1^)	12.92 ± 2.7^b,c,d^	6.19 ± 1.47^a,c,d^	3.79 ± 0.99^a,b,d^	2.56 ± 0.69^a,b,c^
	Decelerations distance (m · min^−1^)	33.16 ± 8.95^b,c,d^	13.7 ± 3.48^a,c,d^	8.56 ± 2.45^a,b,d^	5.43 ± 1.73^a,b,c^
	Sprint duration (s · min^−1^)	17.3 ± 5.6^b,c,d^	6.43 ± 2.25^a,c,d^	3.64 ± 1.48^a,b,d^	2.13 ± 0.92^a,b,c^
	Jumps (n · min^−1^)	4.24 ± 3.32^b,c,d^	1.44 ± 1.18^a,c,d^	0.76 ± 0.63^a,b^	0.41 ± 0.37^a,b^
	Change of directions (n · min^−1^)	8.89 ± 3.02^b,c,d^	3.08 ± 1.09^a,c,d^	1.69 ± 0.6^a,b,d^	0.96 ± 0.38^a,b,c^

**Table:** 

Team	Variable	p-values						Effect Size					

		p (10s–30s)	p (10s–60s)	p (10s–120s)	p (30s–60s)	p (30s–120s)	p (60s–120s)	d (10s–30s)	d (10s–60s)	d (10s–120s)	d (30s–60s)	d (30s–120s)	d (60s–120s)
Male	Distance (m · min^−1^)	< 0.001	< 0.001	< 0.001	< 0.001	< 0.001	< 0.001	3.770	5.877	7.199	2.375	4.199	2.286
	Accelerations count (n · min^−1^)	< 0.001	< 0.001	< 0.001	< 0.001	< 0.001	< 0.001	2.996	4.295	5.076	1.920	3.300	1.598
	Accelerations distance (m · min^−1^)	< 0.001	< 0.001	< 0.001	< 0.001	< 0.001	< 0.001	3.379	4.613	5.370	1.865	3.252	1.614
	Decelerations count (n · min^−1^)	< 0.001	< 0.001	< 0.001	< 0.001	< 0.001	< 0.001	3.063	4.294	5.106	1.867	3.307	1.528
	Decelerations distance (m · min^−1^)	< 0.001	< 0.001	< 0.001	< 0.001	< 0.001	< 0.001	3.168	4.293	4.986	1.811	3.168	1.562
	Sprint duration (s · min^−1^)	< 0.001	< 0.001	< 0.001	< 0.001	< 0.001	< 0.001	2.969	4.046	4.659	1.563	2.694	1.547
	Jumps (n · min^−1^)	< 0.001	< 0.001	< 0.001	< 0.001	< 0.001	< 0.001	1.631	2.224	2.505	1.026	1.641	0.861
	Change of directions (n · min^−1^)	< 0.001	< 0.001	< 0.001	< 0.001	< 0.001	0.002	2.228	2.943	3.296	1.507	2.463	1.411

Female	Distance (m · min^−1^)	< 0.001	< 0.001	< 0.001	< 0.001	< 0.001	< 0.001	3.479	5.081	6.473	2.207	4.232	2.094
	Accelerations count (n · min^−1^)	< 0.001	< 0.001	< 0.001	< 0.001	< 0.001	< 0.001	3.745	5.390	6.397	2.211	3.739	1.589
	Accelerations distance (m · min^−1^)	< 0.001	< 0.001	< 0.001	< 0.001	< 0.001	< 0.001	3.091	4.309	4.901	1.887	3.030	1.505
	Decelerations count (n · min^−1^)	< 0.001	< 0.001	< 0.001	< 0.001	< 0.001	< 0.001	3.097	4.490	5.254	1.918	3.160	1.435
	Decelerations distance (m · min^−1^)	< 0.001	< 0.001	< 0.001	< 0.001	< 0.001	< 0.001	2.865	3.748	4.300	1.708	3.009	1.475
	Sprint duration (s · min^−1^)	< 0.001	< 0.001	< 0.001	< 0.001	< 0.001	0.001	2.548	3.337	3.782	1.466	2.502	1.221
	Jumps (n · min^−1^)	< 0.001	< 0.001	< 0.001	0.012	< 0.001	0.185	1.125	1.454	1.621	0.709	1.170	0.683
	Change of directions (n · min^−1^)	< 0.001	< 0.001	< 0.001	< 0.001	< 0.001	0.003	2.562	3.312	3.692	1.575	2.595	1.468

The super-indices indicate that the mixed model reveals the existence of differences (p < 0.05) between a: 10 s, b: 30 s, c: 1 min, d: 2 min.

For females, the relative distances covered (m · min^−1^) decreased from 237.37 ± 24.16 m · min^−1^ over 10 s to 114.52 ± 11.7 m · min^−1^ over 120 s (p < 0.001), showing a nearly perfect effect size (d = 6.47). The relative number of accelerations and decelerations (n · min^−1^) also decreased, from 12.99 ± 2.24 n · min^−1^ to 2.44 ± 0.65 n · min^−1^ for accelerations (p < 0.001; d = 6.40, nearly perfect), and from 12.92 ± 2.7 n · min^−1^ to 2.56 ± 0.69 n · min^−1^ for decelerations (p < 0.001; d = 5.25, nearly perfect). However, for female players, the relative PD for jumps did not show a significant difference between the 60 s and 120 s epochs (0.76 ± 0.63 vs. 0.41 ± 0.37; p = 0.185, d = 0.68).

### Player Position Differences

The comparison of PD between FC and BC is detailed in Table 2. For males, BC generally recorded higher values across most physical demand variables. For instance, in the 60 s window, they covered more total distance than FC (144.83 ± 11.95 m vs. 139.53 ± 10.72 m; p = 0.002) with a small effect size (d = -0.46). Similarly, they performed more accelerations (4.89 ± 1.07 vs. 4.18 ± 0.97; p < 0.001) with a moderate effect size (d = -0.70), and more decelerations (4.78 ± 1.11 vs. 3.78 ± 0.91; p < 0.001) with a moderate effect size as well (d = -0.97). These positional differences persisted across the 10 s, 30 s, and 120 s time windows.

**TABLE 2:** Peak demands between players position over different time epochs (mean ± SD)

Team	Variable	Window							

		10 s				30 s			

		Frontcourt	Backcourt	*p*	Effect Size	Frontcourt	Backcourt	*p*	Effect Size
**Male**	Distance (m) ^a,b,c,d^	40.69 ± 3.56	42.83 ± 3.93	< 0.001	-0.568	85.67 ± 7.09	89.58 ± 8.25	0.001	-0.503
	Accelerations (count) ^a,b,c,d^	2.28 ± 0.51	2.55 ± 0.5	< 0.001	-0.544	3.35 ± 0.77	3.78 ± 0.77	< 0.001	-0.570
	Accelerations (m) ^a,b,c,d^	5.95 ± 1.41	6.74 ± 1.21	< 0.001	-0.611	7.56 ± 2.12	8.94 ± 1.65	< 0.001	-0.738
	Decelerations (count) ^a,b,c,d^	2.26 ± 0.44	2.42 ± 0.52	0.031	-0.334	3.15 ± 0.68	3.63 ± 0.77	< 0.001	-0.662
	Decelerations (m) ^a,b,c,d^	6.06 ± 1.49	6.85 ± 1.43	< 0.001	-0.546	7.42 ± 1.66	9.11 ± 2.08	< 0.001	-0.886
	Sprint duration (s) ^a,b,c,d^	3.36 ± 0.85	3.76 ± 0.97	0.007	-0.425	3.78 ± 1.13	4.46 ± 1.51	0.001	-0.500
	Jump (count) ^a,b,d^	1.33 ± 0.66	1.53 ± 0.66	0.047	-0.248	1.35 ± 0.67	1.58 ± 0.87	0.017	-0.288
	Changes of direction (count)	1.74 ± 0.62	1.67 ± 0.66	0.488	0.108	1.85 ± 0.72	1.91 ± 0.7	0.625	-0.076

**Female**	Distance (m) ^b^	38.9 ± 3.84	40.51 ± 4.15	0.067	-0.406	82.82 ± 7.15	86.69 ± 6.02	0.012	-0.577
	Accelerations (count)	2.18 ± 0.39	2.14 ± 0.36	0.69	0.099	3.1 ± 0.68	3.09 ± 0.56	0.88	0.023
	Accelerations (m) ^c,d^	4.91 ± 1.32	5.39 ± 1.03	0.07	-0.399	6.19 ± 1.93	6.77 ± 1.45	0.130	-0.330
	Decelerations (count) ^d^	2.1 ± 0.46	2.23 ± 0.43	0.20	-0.287	2.98 ± 0.77	3.26 ± 0.66	0.08	-0.382
	Decelerations (m) ^a,b,d^	5.16 ± 1.44	6.05 ± 1.42	0.01	-0.618	6.51 ± 1.81	7.34 ± 1.53	0.03	-0.489
	Sprint duration (s)	2.77 ± 0.92	3.05 ± 0.94	0.24	-0.308	3.09 ± 1.04	3.4 ± 1.23	0.24	-0.278
	Jump (count) ^d^	0.76 ± 0.59	0.63 ± 0.49	0.28	0.238	0.8 ± 0.64	0.6 ± 0.5	0.11	0.342
	Changes of direction (count)	1.44 ± 0.5	1.54 ± 0.51	0.35	-0.204	1.48 ± 0.54	1.63 ± 0.55	0.22	-0.273


**Team**	**Variable**	**Window**							

		**60 s**				**120 s**			

		**Frontcourt**	**Backcourt**	***p***	**Effect Size**	**Frontcourt**	**Backcourt**	***p***	**Effect Size**
**Male**	Distance (m) ^a,b,c,d^	139.53 ± 10.72	144.83 ± 11.95	0.002	-0.464	222.03 ± 26.25	231.27 ± 27.4	0.01	-0.346
	Accelerations (count) ^a,b,c,d^	4.18 ± 0.97	4.89 ± 1.07	< 0.001	-0.701	5.73 ± 1.42	6.56 ± 1.42	< 0.001	-0.575
	Accelerations (m) ^a,b,c,d^	9.23 ± 2.59	11.21 ± 2.44	< 0.001	-0.788	12.06 ± 3.38	14.39 ± 3.23	< 0.001	-0.706
	Decelerations (count) ^a,b,c,d^	3.78 ± 0.91	4.78 ± 1.11	< 0.001	-0.967	5.03 ± 1.28	6.27 ± 1.5	< 0.001	-0.889
	Decelerations (m) ^a,b,c,d^	8.92 ± 2.34	11.34 ± 2.77	< 0.001	-0.936	11.14 ± 3.06	14.53 ± 3.54	< 0.001	-1.017
	Sprint duration (s) ^a,b,c,d^	4.39 ± 1.19	5.07 ± 1.83	0.006	-0.424	5.04 ± 1.39	5.84 ± 2.04	0.004	-0.450
	Jump (count) ^a,b,d^	1.53 ± 0.81	1.73 ± 0.93	0.06	-0.226	1.85 ± 0.97	2.13 ± 0.82	0.030	-0.248
	Changes of direction (count)	1.95 ± 0.74	2.1 ± 0.76	0.084	-0.269	2.3 ± 0.89	2.45 ± 0.85	0.25	-0.180

**Female**	Distance (m) ^b^	137.88 ± 13.05	142.48 ± 11.09	0.11	-0.374	227.6 ± 24.63	231.07 ± 21.69	0.61	-0.148
	Accelerations (count)	3.64 ± 1.01	3.86 ± 0.85	0.34	-0.230	4.8 ± 1.39	5.0 ± 1.19	0.53	-0.153
	Accelerations (m) ^c,d^	7.01 ± 2.13	7.96 ± 1.87	0.03	-0.467	9.07 ± 2.83	10.26 ± 2.28	0.04	-0.452
	Decelerations (count) ^d^	3.66 ± 1.02	3.97 ± 0.92	0.15	-0.317	4.88 ± 1.47	5.49 ± 1.17	0.04	-0.448
	Decelerations (m) ^a,b,d^	8.16 ± 2.78	9.12 ± 1.77	0.07	-0.399	10.12 ± 3.9	11.9 ± 2.39	0.02	-0.529
	Sprint duration (s)	3.49 ± 1.52	3.84 ± 1.42	0.33	-0.234	3.96 ± 1.82	4.67 ± 1.84	0.10	-0.386
	Jump (count) ^d^	0.84 ± 0.68	0.66 ± 0.54	0.18	0.292	1.0 ± 0.81	0.57 ± 0.56	0.01	0.598
	Changes of direction (count)	1.62 ± 0.64	1.8 ± 0.53	0.17	-0.303	1.8 ± 0.76	2.09 ± 0.74	0.07	-0.381

The super-indices indicate that the mixed model reveals the existence of differences (p < 0.05) between the frontcourt and backcourt, for the windows a: 10 s, b: 30 s, c: 1 min, d: 2 min.

For females, BC demonstrated higher values for total distance covered in the 30 s window (86.69 ± 6.02 m vs. 82.82 ± 7.15 m; p = 0.012, d = -0.58), acceleration distance over 120 s (10.26 ± 2.28 m vs. 9.07 ± 2.83 m; p = 0.04, d = -0.45), and deceleration distance over 120 s (11.9 ± 2.39 m vs. 10.12 ± 3.9 m; p = 0.02, d = -0.53). In contrast, FC showed superior results for the number of jumps in the 120 s window (1.0 ± 0.81 vs. 0.57 ± 0.56; p = 0.01, d = 0.60).

### Characterization of High Intensity Period and Very High Intensity Period

For male players, FC were more frequently exposed to HIP over 10 s for the distance covered, reflecting a trivial difference (7.62 ± 4.68 vs. 6.71 ± 4.38; p = 0.011, d = 0.20; Table 3). Small-effect emerged over 30 s for both HIP (8.0 ± 4.6 vs. 6.86 ± 4.25; p = 0.001, d = 0.26) and VHIP (3.16 ± 2.10 vs. 2.65 ± 1.87; p = 0.001, d = 0.26). Conversely, BC were more frequently exposed to HIP (1.07 ± 1.02 vs. 0.80 ± 0.84; p < 0.001, d = -0.29) and VHIP (1.34 ± 0.70 vs. 1.17 ± 0.61; p = 0.001, d = -0.25) over 30 s during high-intensity deceleration, both reflecting small effect sizes. A similar trend emerged over 120 s for both HIP (1.57 ± 1.36 vs. 1.08 ± 1.14; p < 0.001, d = -0.38) and VHIP (1.34 ± 0.83 vs. 1.17 ± 0.76; p = 0.015, d = -0.21).

**Table 3:** Frequency (mean ± SD) of high intensity and very high intensity periods for male players

Window	Variable	High Intensity Periods						Very High Intensity Periods					

		Frontcourt		Backcourt				Frontcourt		Backcourt	

		80–90%	Count	80–90%	Count	*p*	Effect Size	> 90%	Count	> 90%	Count	*p*	Effect Size
**10 s**	Distance (m)	28.51–32.07	7.62 ± 4.68*	29.71–33.43	6.71 ± 4.38	0.011	0.2	32.07	2.69 ± 1.76	33.43	2.51 ± 1.68	0.206	0.1
	Acceleration (m)	3.64–4.09	0.87 ± 0.9	4.07–4.58	0.97 ± 1.06	0.234	-0.1	4.09	1.3 ± 0.65	4.58	1.39 ± 0.71	0.068	-0.14
	Deceleration (m)	3.65–4.11	0.84 ± 0.93	4.12–4.63	0.88 ± 0.94	0.587	-0.04	4.11	1.35 ± 0.67	4.63	1.34 ± 0.71	0.937	0.01
	Sprint duration (s)	1.89–2.12	0.81 ± 0.98	2.06–2.31	0.78 ± 0.85	0.647	0.03	2.12	1.18 ± 0.67	2.31	1.17 ± 0.6	0.786	0.02

**30 s**	Distance (m)	60.3–67.84	8.0 ± 4.6*	63.09–70.98	6.86 ± 4.25	0.001	0.26	67.84	3.16 ± 2.1^†^	70.98	2.65 ± 1.87	0.001	0.26
	Acceleration (m)	4.42–4.97	1.0 ± 1.08	5.34–6.01	1.21 ± 1.17*	0.019	-0.18	4.97	1.28 ± 0.65	6.01	1.3 ± 0.68	0.797	-0.02
	Deceleration (m)	4.38–4.92	0.8 ± 0.84	5.33–6.0	1.07 ± 1.02*	< 0.001	-0.29	4.92	1.17 ± 0.61	6.0	1.34 ± 0.7 †	0.001	-0.25
	Sprint duration (s)	2.02–2.27	0.7 ± 0.92	2.3–2.59	0.72 ± 0.8	0.738	-0.03	2.27	1.06 ± 0.6	2.59	1.06 ± 0.55	0.907	0.01

**60 s**	Distance (m)	97.54–109.73	5.16 ± 3.66	100.89–113.5	4.97 ± 3.61	0.593	0.05	109.73	2.42 ± 1.57	113.5	2.36 ± 1.61	0.632	0.04
	Acceleration (m)	5.25–5.91	1.16 ± 1.11	6.62–7.45	1.23 ± 1.18	0.530	-0.06	5.91	1.31 ± 0.73	7.45	1.27 ± 0.65	0.335	0.06
	Deceleration (m)	5.08–5.71	1.02 ± 1.02	6.59–7.41	1.23 ± 1.13*	0.017	-0.19	5.71	1.22 ± 0.58	7.41	1.27 ± 0.7	0.367	-0.07
	Sprint duration (s)	2.26–2.54	0.66 ± 0.84	2.59–2.91	0.79 ± 0.91*	0.020	-0.16	2.54	0.95 ± 0.52	2.91	1.02 ± 0.52	0.086	-0.13

**120 s**	Distance (m)	152.09–171.1	4.66 ± 3.36	156.18–175.7	4.32 ± 3.71	0.207	0.09	171.1	2.28 ± 1.8	175.7	2.15 ± 1.75	0.281	0.08
	Acceleration (m)	6.86–7.72	1.39 ± 1.3	8.51–9.57	1.62 ± 1.51*	0.032	-0.16	7.72	1.21 ± 0.67	9.57	1.31 ± 0.85	0.110	-0.13
	Deceleration (m)	6.36–7.15	1.08 ± 1.14	8.55–9.61	1.57 ± 1.36*	< 0.001	-0.38	7.15	1.17 ± 0.76	9.61	1.34 ± 0.83^†^	0.015	-0.21
	Sprint duration (s)	2.6–2.93	0.74 ± 0.85	3.01–3.39	0.88 ± 0.95	0.068	-0.16	2.93	0.94 ± 0.56	3.39	0.97 ± 0.59	0.569	-0.05

The super-indices indicate that the mixed model reveals the existence of differences (p < 0.05) between frontcourt and backcourt, for high-intensity scenario:

* and very-high intensity scenario: †.

Among female players, BC consistently demonstrated more frequent passages in VHIP for sprint durations across all time windows, with these differences classified as small effects. Specifically, BC recorded higher values than FC at 10 s (1.19 ± 0.60 vs. 1.02 ± 0.62; p = 0.023, d = -0.27), 30 s (1.16 ± 0.64 vs. 0.92 ± 0.57; p < 0.001, d = -0.40), 60 s (1.11 ± 0.53 vs. 0.92 ± 0.60; p = 0.003, d = -0.34), and 120 s (0.94 ± 0.52 vs. 0.81 ± 0.57; p = 0.036, d = -0.23; Table 4).

**Table 4:** Frequency (mean ± SD) of high intensity and very high intensity periods for female players

Window	Variable	High Intensity Periods						Very High Intensity Periods					

		Frontcourt		Backcourt				Frontcourt		Backcourt	

		80–90%	Count	80–90%	Count	*p*	Effect Size	> 90%	Count	> 90%	Count	*p*	Effect Size
**10 s**	Distance (m)	27.73–31.2	7.57 ± 5.0	28.62–32.19	8.19 ± 4.71	0.243	-0.13	31.2	2.64 ± 1.71	32.19	3.12 ± 2.09†	0.024	-0.25
	Acceleration (m)	2.94–3.31	0.9 ± 1.1	3.29–3.7	0.84 ± 0.89	0.647	0.06	3.31	1.32 ± 0.68	3.7	1.29 ± 0.59	0.683	0.05
	Deceleration (m)	3.09–3.47	0.75 ± 0.93	3.63–4.08	0.74 ± 0.92	0.945	0.01	3.47	1.34 ± 0.68	4.08	1.34 ± 0.66	0.782	0.01
	Sprint duration (s)	1.46–1.64	0.65 ± 0.82	1.73–1.94	0.79 ± 0.84	0.266	-0.16	1.64	1.02 ± 0.62	1.94	1.19 ± 0.6†	0.023	-0.27

**30 s**	Distance (m)	59.28–66.69	8.04 ± 4.97	61.97–69.71	7.51 ± 4.4	0.572	0.11	66.69	2.96 ± 1.93	69.71	2.78 ± 1.89	0.444	0.09
	Acceleration (m)	3.61–4.06	0.9 ± 1.02	4.11–4.63	0.81 ± 0.84	0.432	0.1	4.06	1.28 ± 0.73	4.63	1.23 ± 0.65	0.503	0.08
	Deceleration (m)	3.83–4.3	0.74 ± 0.78	4.47–5.03	0.94 ± 0.86*	0.042	-0.23	4.3	1.26 ± 0.64	5.03	1.28 ± 0.57	0.657	-0.03
	Sprint duration (s)	1.56–1.75	0.57 ± 0.76	1.85–2.08	0.69 ± 0.81	0.197	-0.14	1.75	0.92 ± 0.57	2.08	1.16 ± 0.64†	< 0.001	-0.4

**60 s**	Distance (m)	96.95–109.07	6.02 ± 3.99	100.89–113.5	5.94 ± 3.87	0.460	0.02	109.07	2.77 ± 1.87	113.5	2.79 ± 1.78	0.762	-0.01
	Acceleration (m)	4.25–4.78	1.09 ± 1.18	4.83–5.43	0.91 ± 0.94	0.159	0.17	4.78	1.34 ± 0.78	5.43	1.23 ± 0.56	0.178	0.15
	Deceleration (m)	4.6–5.17	0.92 ± 1.0	5.59–6.28	1.17 ± 1.0*	0.002	-0.26	5.17	1.18 ± 0.64	6.28	1.31 ± 0.68	0.056	-0.2
	Sprint duration (s)	1.66–1.87	0.55 ± 0.73	2.09–2.35	0.67 ± 0.79	0.167	-0.16	1.87	0.92 ± 0.6	2.35	1.11 ± 0.53†	0.003	-0.3

**120 s**	Distance (m)	156.52–176.09	4.47 ± 3.64	163.23–183.63	4.52 ± 3.8	0.225	-0.01	176.09	2.25 ± 1.89	183.63	2.32 ± 1.96	0.408	-0.04
	Acceleration (m)	5.51–6.2	1.18 ± 1.21	6.13–6.9	1.26 ± 1.2	0.472	-0.06	6.2	1.21 ± 0.69	6.9	1.31 ± 0.62	0.165	-0.15
	Deceleration (m)	5.63–6.33	1.2 ± 1.22	7.14–8.03	1.38 ± 1.22	0.133	-0.15	6.33	1.18 ± 0.8	8.03	1.27 ± 0.74	0.275	-0.12
	Sprint duration (s)	1.89–2.13	0.5 ± 0.74	2.47–2.78	0.64 ± 0.86	0.127	-0.17	2.13	0.81 ± 0.57	2.78	0.94 ± 0.52†	0.036	-0.23

The super-indices indicate that the mixed model reveals the existence of differences (p < 0.05) between frontcourt and backcourt, for high-intensity scenario:

* and very-high intensity scenario: †.

## DISCUSSION

This study is the first to provide a comprehensive characterization of MDS in elite youth basketball players. In alignment with the stated aims, the study investigated MDS subcategories (PD, HIP and VHIP) across both male and female athletes and explored how these varied by playing position. The findings revealed three key outcomes: (1) shorter time epochs consistently captured higher PD values for both male and female players; (2) BC, generally exhibited greater PD than FC; and (3) male FC were more frequently exposed in HIP and VHIP in terms of distance covered, whereas female BC consistently displayed longer and more frequent sprint durations during VHIP. These results provide valuable references for developing targeted, position-specific training programs and inform strategies to prepare athletes for the most challenging phases of competition.

### Time Epoch Impact

The results showed that shorter time epochs capture significantly higher PD across all physical variables, which aligns with previous research on the MDS in sports [7, 9, 36]. For example, Alonso et al., [7] found significant differences in Player Load (PL) between 1-minute, 5-minute, 10-minute, and full-game periods for youth elite male basketball players. Additionally, Fleureau et al., [36] observed similar trends in elite handball players, noting that the reduction in intensity over time follows a power law, supporting the importance of focusing on shorter, high-intensity periods. These differences in PD across different time windows may be attributed to fatigue-related mechanisms, as evidenced by prior research indicating that factors such as glycogen depletion and muscle damage contribute to a reduction in external demands across basketball games [37]. In shorter epochs, players can still rely on readily available energy stores and minimal muscle fatigue, thereby maintaining higher intensity levels, while longer epochs see a gradual decline in performance due to the progressive depletion of energy reserves and increased muscular strain. These results underscore the intermittent nature of basketball, supporting the need for training programs that focus on short, intense bursts to mimic match demands.

### Player Position Differences

The findings also highlight significant position-dependent differences in physical demands, with male BC consistently exhibiting higher peak values across all measures and time epochs than FC. This finding aligns with García et al. [12], who reported that centers covered the lowest peak total distance, while forwards showed the greatest peak distances at speeds > 18 km/h over a 1-minute window, reflecting the higher mobility and activity demands on guards. In female players, a similar trend in distance covered was only observed over a 30-second window, potentially due to differing game tempos, play styles, and physiological attributes. Female players may distribute physical effort more evenly, leading to fewer high-intensity peaks over short intervals, although trends were observed across other time windows as well. Further research is needed to confirm these differences.

The present study also shows a greater number of high-intensity accelerations for male BC, consistent with trends in professional basketball [12] and elite under-18 teams [13]. This reinforces the frequent high-intensity acceleration and deceleration demands on BC in comparison to FC positions.

While males showed significant position-based differences across almost all variables and time windows, the distinctions among females were more nuanced. Prior studies, such as Delextrat et al. [38], have observed certain positional differences in female players, with point guards, for instance, showing more movements per minute and more sprints compared to power forwards and centers. In our study, female FC performed more jumps than BC over the 120-second window, aligning with their roles in rebounding and shot-blocking [38]. However, the absence of studies investigating MDS in female basketball limits further comparisons, emphasizing a need for more research to better understand positional differences in this context. The current findings reinforce the distinct physical demands tied to each position: BC, often tasked with ball handling and quick transitions, engage in frequent high-intensity actions, whereas FC, typically involved in close-range positional play, demonstrate different demands [39]. These insights support position-specific training strategies to optimize performance.

### Characterization of High Intensity Period and Very High-Intensity Period

Finally, this study characterizes HIP and VHIP frequencies in elite youth basketball, revealing once again positional differences for both male and female players. Male FC were more frequently exposed to HIP and VHIP for the distance covered over 10- and 30-second windows. The evolving role of centers, who now demonstrate improved coordination and quick movements across various spatial contexts, may explain this result [40]. Another possible explanation is that they often exhibit lower PD, which gives them more opportunities to exceed 80% and 90% of their peak values. Because they frequently perform full-court runs from basket to basket [41], their movement patterns tend to be more continuous and less prone to abrupt spikes in intensity, thereby allowing them to sustain relatively higher intensities more consistently.

Conversely, BC are more frequently exposed to HIP and VHIP during high-intensity deceleration over 30- and 120-second intervals. This is consistent with existing literature, which shows that guards typically perform more high-intensity decelerations (> 3 m · s^−2^) due to their positional roles, as they often face numerous picks, hand-offs, and screens to create scoring opportunities on the perimeter [42, 43]. Furthermore, this pattern aligns with the physical principle that lighter, more agile guards can change speed more rapidly, enabling them to execute more high-intensity actions than their heavier FC counterparts [44].

Notably, our findings differ from the only other study that assessed positional differences in match demands relative to PD in male players, which found minimal differences across positions for periods above 80% intensity [22]. This discrepancy may stem from methodological differences: García et al., used only 30- and 60-second epochs within one male team, while our study included both genders and additional time windows (10, 30, 60, and 120 seconds). Our broader approach thus offers a more comprehensive view of MDS intensity and frequency.

Additionally, female BC demonstrated more frequent passages in VHIP for sprint durations across all time windows. This aligns with a previous study showing that guards performed more sprints than bigs in the Spanish National Division 1 (Liga Femenina 1) [38]. While this prior research is the closest to our current observations, direct comparisons remain difficult, as no other study has specifically examined MDS in female basketball. Nevertheless, these positional differences in HIP and VHIP frequencies reinforce the need for targeted conditioning programs. Training regimens tailored to each position’s specific demands could enhance performance, ultimately benefiting team success.

### Limits and perspectives

While this study is the first to categorize MDS into distinct subcategories for male and female elite youth basketball players, several limitations should be acknowledged. A primary limitation is the relatively small sample size (31 players: 20 males, 11 females), which, though providing valuable insights, limits the generalizability of findings. Future studies should aim for larger, more diverse samples to validate these results across different teams and leagues. Additionally, as data were collected exclusively from home games, potential biases may exist in external load measurements [45, 46]. Despite the insights gained, there remain many unexplored aspects of MDS in basketball, particularly around WCS. WCSs are complex events marked by extreme internal responses triggered by a combination of physical exertion, psychological stress, environmental factors, and tactical demands [11]. These scenarios offer an additional dimension to understanding MDS, capturing instances where players operate at their absolute limits. Therefore, future research should consider the analysis of WCS to obtain a better understanding of the MDS during basketball competition and to consequently optimise individual player preparation.

### Practical implications

This study offers valuable insights for coaches and sports scientists working with elite youth basketball players, particularly regarding the position-specific physical demands encountered during MDS. The results indicate that BC experience higher PD across all measured variables, suggesting the need to emphasize agility and high-intensity running in their training. Conversely, FC—especially in male teams—cover greater distances during HIP and VHIP, underscoring the importance of conditioning strategies aimed at enhancing their capacity to sustain high-intensity efforts.

To help players maintain performance quality throughout these demanding periods, practitioners can incorporate isolated running drills, such as short intervals or repeated sprints, which specifically target the movement patterns and energy systems central to MDS. However, it is crucial to note that, as highlighted in a recent study on the frequency of MDS in U19 professional football [47], MDS should not be used as direct benchmarks for replicating exercises but rather as a basis for prescribing appropriate training stimuli.

Additionally, while the use of LPS technology facilitated comprehensive locomotor tracking in this study, teams without access to such resources can rely on the findings presented in Tables 1 to 4. These data can serve as position- and gender-specific references, guiding coaches in devising training programs that help athletes better cope with the stressors encountered during competition.

## CONCLUSIONS

This study provides a detailed analysis of MDS in elite youth basketball, highlighting the sport’s unique physical demands. By examining various time epochs and player positions, the findings indicate that shorter time windows capture higher intensity demands, reflecting basketball’s intermittent and high-intensity nature. Notably, significant differences were observed between frontcourt and backcourt players, emphasizing the need for position-specific training programs. Furthermore, the characterization of HIP and VHIP offers valuable insights into the physical challenges players face during competition. This study is the first to include both male and female players, thereby advancing the understanding of specific physical demands in female basketball. These findings hold practical relevance for coaches and sports scientists seeking to develop training strategies that optimize performance.
